# Effect of Taro Starch, Beet Juice, Probiotic, and/or Psicose on Gut Microbiota in a Type 2 Diabetic Rat Model: A Pilot Study

**DOI:** 10.1155/2021/1825209

**Published:** 2021-05-20

**Authors:** Ingrid S. Surono, Ata Aditya Wardana, Priyo Waspodo, Budi Saksono, Koen Venema

**Affiliations:** ^1^Food Technology Department, Faculty of Engineering, Bina Nusantara University, Jakarta 11480, Indonesia; ^2^Research Center for Biotechnology, Lembaga Ilmu Pengetahuan Indonesia, Jalan Raya Bogor Km 46, Cibinong 16911, Indonesia; ^3^Maastricht University-Campus Venlo, Centre for Healthy Eating & Food Innovation (HEFI), Villafloraweg 1, 5928 SZ Venlo, Netherlands

## Abstract

*Background and Objectives*. The gut microbiota has been shown to be involved in the development and severity of type 2 diabetes (T2D). The aim of the present study was to test the effect of potential functional food ingredients, alone or in combination, on the gut microbiota composition in diabetic rats in a pilot study of 1 week of feeding. *Methods*. In a pilot study to modulate the composition of the gut microbiota, (i) native taro starch, (ii) modified taro starch, (iii) beet juice, (iv) psicose, (v) the probiotic *L. plantarum* IS-10506, (vi) native starch combined with beet juice, (vii) native starch to which beet juice was adsorbed, (viii) modified starch combined with beet juice, and (ix) modified starch to which beet juice was adsorbed were fed to rats in which T2D was induced with streptozotocin (STZ). After one week, the composition of the gut microbiota was evaluated by sequencing the PCR-amplified V3-V4 region of the 16S rRNA gene. *Results and Conclusions*. The next-generation sequencing showed that 13 microbial taxa of the gut microbiota were significantly different between groups, depending on the treatment. The results of this pilot study will be used to design a 4-week intervention study in STZ-induced T2D rats to determine the best functional food for counteracting T2D, including their effects on satiety hormones. This should ultimately lead to the development of functional foods for prediabetic and diabetic individuals.

## 1. Introduction

The global incidence of T2D is predicted to reach 578 million cases by the year 2030 and 700 million cases by 2045, a predicted increase of 51% from the 463 million cases in 2019[1, 2]. Besides an increase in energy intake and a decrease in energy expenditure contributing to obesity and T2D, the gut microbiota has also been shown to play a role in the disease [3, 4]. It is common knowledge that the gut microbiota composition and/or activity can be changed using food components [5]. The vital role of food for prevention and treatment of T2D needs proper attention, such as in the development of dietary components that positively influence postprandial glycaemia and through this their potential to reduce the impact of T2D, or components that function through the gut microbiota [3, 4, 6, 7]. Such dietary components could be taro, psicose, probiotics, and beetroot as indicated in the subsequent sections.

In Indonesia, taro (Cocoyam) is cultivated and traditionally used as food crop by several ethnic communities in Borneo [8], and its utilization is also related to the culture of a region; hence, taro is very important for ethnic community-life [9]. Kreike et al. [10] reported that Indonesia has the highest taro diversity in the world and apart from Borneo it is found in areas in Java, Sumatra, and Sulawesi [11]. Taro is used as an alternative carbohydrate source to reduce dependence on rice. Processing taro into flour can also be an alternative substitute for wheat flour, which has been widely used but depends on imports. Furthermore, taro use increases efforts to diversify food and support food security. Taro contains 41% resistant starch (RS) [12], which reaches the colon and can contribute to the modulation of the gut microbiota and increases the amount of the health-promoting microbial metabolite butyrate [13]. Taro starch can be modified to increase the amount of RS [14].

Another functional dietary component that has been shown to affect T2D is D-psicose (or D-allulose), a rare monosaccharide. In an attempt to produce functional foods aimed at low calorie and less sugar intake for T2D, D-psicose is considered as a substitute for sugar with proven antihyperglycemic, antihyperlipidemic, and anti-inflammatory effects.

Also probiotics have been shown to be beneficial in treatment of T2D [15]. Probiotics are life microorganisms, which when administered in adequate amounts have a beneficial effect on the host [16, 17]. The traditional fermented buffalo milk, dadih, produced in West Sumatra [18] has been shown to reduce adiposity, weight gain, and adiposity inflammation in high fat induced obese rats [19]. Probiotic strains have been isolated from dadih, including *L. plantarum* IS-10506 [20, 21].

A last functional class of dietary components considered to be beneficial in T2D is polyphenols. Polyphenols interact heavily with the gut microbiota, either because they have been shown to inhibit certain species or on the other side because they stimulate other taxa and are converted in other bioactive phenolic components [22]. Polyphenols including flavonoids, phenolic acids, proanthocyanidins, and tannins have been suggested to be able to modify postprandial glycaemia [23, 24]. Polyphenols may alter glycaemia by inhibiting carbohydrate (CHO) digestion, reducing CHO absorption in the intestines, stimulation of insulin release from pancreatic *ß*-cells, modulation of hepatic glucose output, activation of insulin receptors, or modulation of glucose uptake in insulin-sensitive cells [25]. Moreover, polyphenols and related compounds have been described to reduce postprandial hyperglycaemia and prevent reactive hyperinsulinaemia by reducing the digestion, absorption, and transport of glucose [26]. Beetroot juice has received attention containing a number of compounds including phenolic acids, flavonoids, and betalains [27], and beetroot juice has a high total antioxidant capacity and total polyphenol content [28]. Polyphenols are not very well absorbed in the small intestine and reach the colon where they can modulate the gut microbiota.

The aim of the present study was to find out the effect of (modified) taro starch, psicose, the probiotic *L. plantarum* IS-10506, and phytonutrients of beet juice, alone or in combination, on the gut microbiota composition in streptozotocin-induced T2D rats in a pilot study of 1 week of feeding.

## 2. Materials and Methods

Streptozotocin (STZ) was purchased from Enzo Life Sciences (NY, USA). Purified Rodent Diet AIN-93M, a modified AIN-76A standard diet (American Institute of Nutrition) [29], was used as a control.

### 2.1. Animals and Housing

All animal procedures undertaken were approved by Ethics Committee of the Faculty of Medicine, University of Indonesia (Ref:1196/UN2.F1/ETIK/2018). A total of 40 Male Sprague Dawley rats were purchased from Animal Experimental Laboratory, National Agency of Drug and Food Control (Jakarta, Indonesia), at 6 weeks of age and were allowed to adapt for 14 days. They were housed in individual cages and maintained at 21–23°C and 55% ± 5% humidity with 12-hour light/dark cycle. During the 14 days' acclimatization period, all rodents were given *ad libitum* access to water and commercially available rat normal pellet diet (NPD) purchased from local market, prior to the dietary manipulation.

### 2.2. Development of Type 2 Diabetes by STZ Treatment

Four rats in each group were allocated to the dietary treatments, AIN-93M (AIN), modified AIN-93M by replacing corn starch with taro starch, sucrose and cellulose were replaced with maltodextrin, respectively, for 3 days, with 75, 50, and 25% commercial rat pellet diet and 25, 50, and 75% dietary intervention formulation, respectively. Then the rats were injected intraperitoneally (i.p.) with 120 mg kg^−1^ nicotinamide in 0.9% NaCl, followed after 15 minutes by STZ in citrate buffer pH 4.4 (70 mg kg^−1^), and, four days after the STZ injection, fasting and postprandial blood glucose levels were measured using a freestyle glucose meter (Easy Touch GCU 3 in 1) from tip of the tail. The rats with a fasting glucose of >100 mg dl^−1^ and/or postprandial blood glucose levels of ≥140 mg dl^−1^ were considered as type 2 diabetic, and those rats which had not yet developed T2D within these 4 days were injected for the second time with 120 mg kg^−1^ nicotinamide and STZ in citrate buffer pH 4.4 (70 mg kg^−1^). After confirmation of T2D, the eight-week-old 190–220 g rats were divided into 10 groups of *n* = 4 each, namely, (i) AIN-93M (control), (ii) AIN-93M with two times in a day 3 ml psicose by gavage, (iii) AIN-93M with two times in a day 3 ml beetroot juice by gavage, (iv) native taro starch, (v) modified taro starch, (vi) native taro starch with beetroot juice adsorbed and then dried, (vii) modified taro starch with beetroot juice adsorbed and then dried, (viii) native taro starch combined with two times in a day 3 ml beetroot juice by gavage, (ix) modified taro starch combined with two times in a day 3 ml beetroot juice by gavage, and (x) *L. plantarum* IS-10506. The native or modified taro starch was replacing the corn starch in AIN-93M.

The feed and water intake of the animals were measured. The rats were allowed to continue to feed on their respective diets until the end of the study. Food intake and bodyweight were monitored every day and at the end of one week, respectively, during the study, and the average intake per rat was calculated. Fecal pellets were collected at baseline and at the end of one week of feeding.

### 2.3. Feeding Preparation and Formulation

Taro starch “HASILBUMIKU” was purchased from a local supplier in Bantul, Yogyakarta (Indonesia). Modified taro starch was manufactured by autoclave-cooling according to a modification of the method of Zhao and Lin [14]. In brief, taro starch was blended with distilled water based on the ratio 1 : 3.5, and the blend was then gelatinized using pressure-heated instrument at 121°C for 30 minutes and cooled at 4°C, with a repetition of two cycles. Afterwards, the retrograded starch was dried using a fan-assisted oven at 60°C for 16 hours, after which it was allowed to cool at room temperature for 24 hours and subsequently grounded and sieved using 60 meshes.

Beet juice was adsorbed to both native and modified taro starch by absorbing beetroot juice, at a ratio of 1 : 1, and then drying in an oven at 40°C for 16 hours. These were prepared and fed according to the dose of beet juice of 6 ml/day but in adsorbed beet juice form.

The probiotic was given by gavage at 10^10^ colony forming units (CFU)/day.

### 2.4. Extraction of Nucleic Acids

DNA of feces samples was extracted using the Quick-DNA™ Fecal/Soil Microbe Miniprep Kit (Zymo Research) according to manufacturer's instructions, using the Precellys 24 tissue homogenizer (Bertin Instruments, Montigny-le-Bretonneux, France), applying 3 cycles of 30 seconds each, with 5-minute cooling on ice in between. DNA concentration and purity were checked by measuring absorbance at 260 and 280 nm.

PCR-amplifying the V3-V4 region of the 16S rRNA gene and next-generation sequencing Illumina 16S rRNA gene amplicon libraries were generated and sequenced at BaseClear (Leiden, Netherlands). In short, barcoded amplicons from the V3-V4 region of 16S rRNA genes were generated using a 2-step PCR. 10–25 ng isolated genomic DNA was used as template for the first PCR with a total volume of 50 *μ*l using the 341F (5′-CCTACGGGNGGCWGCAG-3′) and the 785R (5′-GACTACHVGGGTATCTAATCC-3′) primers appended with Illumina adaptor sequences (Illumina, San Diego, CA, USA). PCR products were purified and the sizes of the PCR products were checked on Fragment analyzer (Advanced Analytical Technologies, Heidelberg, Germany) and quantified by fluorometric analysis. Purified PCR products were used for the 2nd PCR in combination with sample-specific barcoded primers (Nextera XT Index Kit, Illumina). Subsequently, PCR products were purified, checked on a Fragment analyzer (Advanced Analytical Technologies), and quantified, followed by multiplexing, clustering, and sequencing on an Illumina MiSeq with the paired-end (2x) 300 bp protocol and indexing.

### 2.5. Sequence Processing and Analyses

The sequencing run was analyzed with the Illumina CASAVA pipeline (v1.8.3) with demultiplexing based on sample-specific barcodes. The raw sequencing data produced was processed removing the sequence reads of too low quality (only “passing filter” reads were selected) and discarding reads containing adaptor sequences or PhiX control with an in-house filtering protocol. A quality assessment on the remaining reads was performed using the FASTQC quality control tool version 0.10.0. Subsequently, the sequences were further analyzed using the Quantitative Insights Into Microbial Ecology (QIIME) software pipeline, version 1.9.1 [30]. Operational taxonomic units (OTUs) were defined at 97% similarity. The measurements for *α*- and *ß*-diversity and visualization of the (un)weighted UniFRac principal coordinate analyses were also done using QIIME. The software package R (3.5.1) [31] was used to determine correlations between OTUs and treatments. Statistical analyses were performed with R (3.5.1) in RStudio (1.0.153). Spearman correlation was calculated between the relative abundance of OTUs and continuous variables (body weight and plasma glucose). Kruskal-Wallis correlations were calculated between OTUs and noncontinuous values (treatments). Multiple comparisons were corrected using the false discovery rate (FDR), and *q*-values (adjusted *p* values) were considered significantly different at <0.05. Correlation between OTUs and continuous values are indicated by the rho-value. Permutational multivariate analysis of variance (PERMANOVA; [32]) was performed to test the significance of *ß*-diversity (weighted and unweighted UniFRac) differences. Hierarchical clustering was performed in R using the function “hclust,” based on the relative abundances of the samples. Dissimilarity values were calculated on the basis of Euclidean distance. Clustering was performed using Ward's minimum variance method.

## 3. Results and Discussion

**Figure 1:**
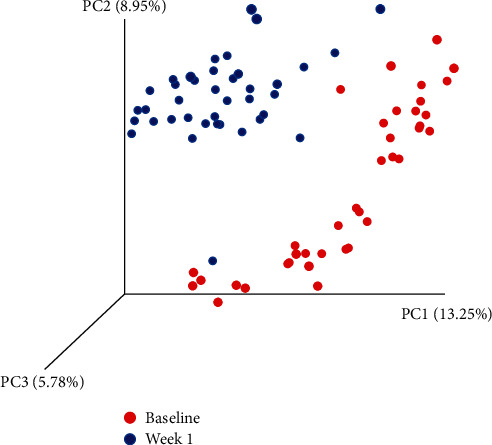
Unweighted principal coordinate analysis of the microbiota composition of the rats at baseline (after induction of T2D) and after one week of feeding the control (AIN) and 9 different treatments.

Compared to baseline (when confirmed T2D was established by STZ injection) the microbiota of the rats was different at week 1 after the interventions with the potential functional food ingredients (displayed as unweighted UniFRac *ß*-diversity in Figure 1 and hierarchical clustering in Figure S1(a)). Figure 1 shows a clear separation of almost all baseline and week 1 samples. Similarly, Figure S1(a) shows hierarchical clustering of most of the samples as well. The *α*-diversity did not differ between groups (measured as Shannon index, observed OTUs, phylogenetic diversity, and evenness; data not shown). Kruskal-Wallis comparison was used to indicate which operational taxonomic units (OTUs) were significantly different between baseline and week 1 (after correction for multiple comparisons, *i.e*., *q* < 0.05). These are listed in Table S1.

**Figure 2:**
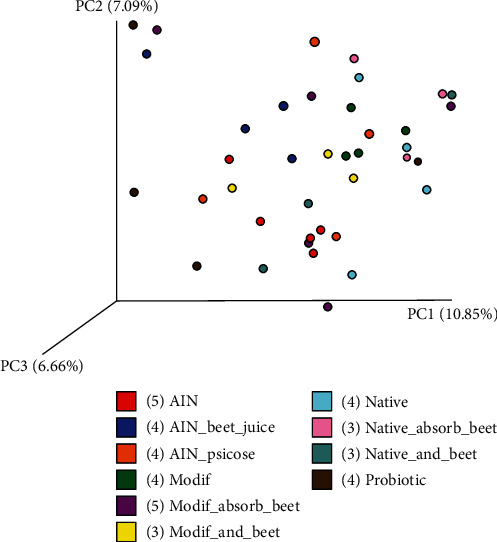
Unweighted principal coordinate analysis of the microbiota composition of the rats after one week of feeding the control (AIN) and 9 different treatments. AIN: control AIN diet; AIN_beet: AIN diet supplemented with beet juice; AIN_Psic: AIN diet supplemented with psicose; Modif: modified taro starch; Mod_absorb_beet: modified taro starch with absorbed beet juice; Modif_and_beet: modified taro starch combined with beet juice by gavage; Native: native taro starch; Nat_absorb_beet: taro starch with absorbed beet juice; Native_and_beet: native taro starch combined with beet juice by gavage; Probiotic: *L.plantarum* IS-10506.

**Figure 3:**
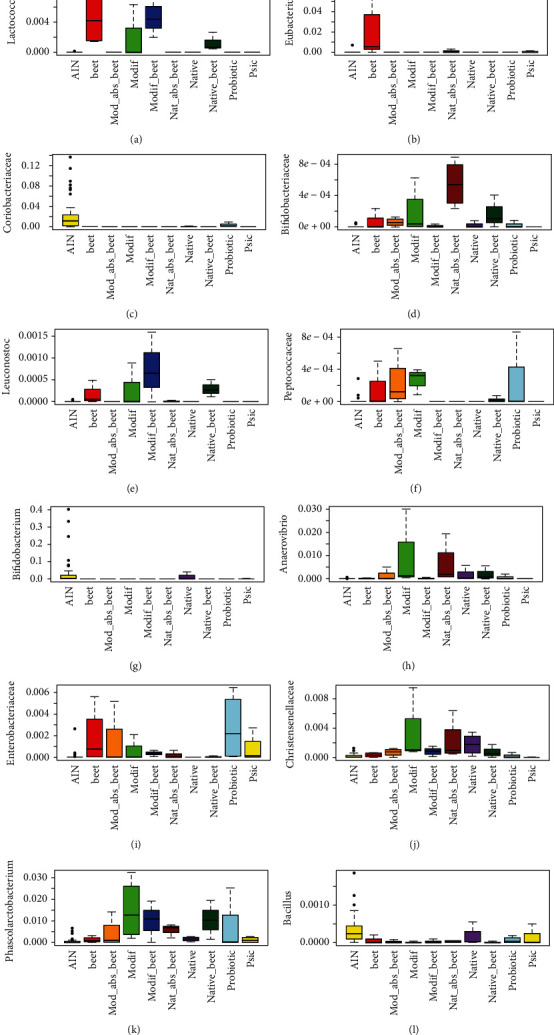
Distribution of the significantly different OTUs (Kruskal-Wallis) with a *q*-value < 0.05 after 1 week of feeding. AIN: control AIN diet; beet: beet juice; Mod_abs_beet: modified taro starch with absorbed beet juice; Modif: modified taro starch; Modif_beet: modified taro starch combined with beet juice by gavage; Nat_abs_beet: taro starch with absorbed beet juice; Native: native taro starch; Native_beet: native taro starch combined with beet juice by gavage; Probiotic: *L.plantarum* IS-10506; Psic: psicose. (a) *Lactococcus*; (b) *Eubacterium*; (c) Coriobacteriaceae; (d) Bifidobacteriaceae; (e) *Leuconostoc*; (f) Peptococcaceae; (g) *Bifidobacterium*; (h) *Anaerovibrio*; (i) Enterobacteriaceae; (j) Christensenellaceae; (k) *Phascolarctobacterium*; (l) *Bacillus*.

After 1 week of dietary intervention, the different interventions did not lead to a clear separation between the groups of rats (Figure 2 and S1(b)).  Figure S1(b) shows color-coding of the samples by whether or not the diets contained taro starch or not. Although there was a better clustering when samples were divided by containing taro starch or not, clustering still was not very strong. This was also still the case when weighted for taxa abundance (not shown). Nevertheless, Kruskal–Wallis comparison between the groups at week 1 displayed that 13 OTUs were significantly (*q* < 0.05) different between groups (Table S2).  Figure 3 shows how these OTUs were distributed over the different treatments. The relative abundance (RA) of Coriobacteriaceae was reduced by all interventions compared to control (AIN). The RA of *Lactococcus*, a lactic acid producing genus, was increased by beet juice, modified starch combined with beet juice, and native starch combined with beet juice. When beet juice was adsorbed to the taro starch, *Lactococcus* was not increased. Acidification of the gut lumen (by lactic acid) is thought to decrease (potential) pathogenic bacteria [33], although this reduction was not observed if one takes Enterobacteriaceae as a marker for potential pathogens (Figure 3). The RA of *Leuconostoc*, another lactic acid producing genus, was increased by similar interventions as for *Lactococcus*, although in addition modified starch showed some increase as well. Beet juice exclusively increased *Eubacterium*, a genus known to produce butyrate, a microbial metabolite considered to be beneficial for the host, as it has been shown to be an important energy source for the colonocytes and has anti-inflammatory effects [34]. The RA of Peptococcaceae was increased by beet juice, modified taro starch, and modified taro starch with absorbed beet juice, in addition to the probiotic *L. plantarum* IS-10506. *Anaerovibrio* was increased by several of the starch interventions. RA of *Bacillus* was reduced by most treatments compared to control, while RA of *Phascolarctobacterium* was increased by all interventions compared to control. The RA of members of the Christensenellaceae was increased by treatment with all taro starch containing interventions. Christensenellaceae has been correlated with leanness, as they have been shown to be enriched in individuals with low body mass index [35]. The genus *Bifidobacterium* seemed to be reduced by all treatments except native starch, while Bifidobacteriaceae were increased by more treatments but particularly by native starch with beet juice (either in combination or adsorbed).

A few OTUs were correlated to body weight (BW) and one OTU was correlated to fasting glucose. Although these were statistically significant even after correction for multiple comparisons (*q* < 0.05), the correlations were rather weak (Table S3; rho values between −0.40 and 0.40).

Being an initial pilot and screening experiment, the study has some inherent limitation. The first is the length of the time of intervention. To allow for an initial quick screening, we chose to feed the functional ingredients for a single week. In the meantime, based on these screening results, we have carried out a study for a 4-week period in rats. Also, based on the results of this study, a clinical trial in prediabetic individuals has been started. In an animal trial, the separate individual animals, coming from the same breeding colony, more or less have the same microbiota composition (although Figure 1 shows that there still is variation between animals at baseline), and, therefore, a group-size of 4 animals per group was used. This was not based on a power-calculation, as it is impossible to predict which microbes would be modulated and, if so, how much. For the recent clinical trial, to account for interindividual variation, we recruited 15 individuals. Other physiological parameters were measured as well, such as several (anti-) inflammatory cytokines and the gut hormones GLP-1 and PYY. We will report separately on these, as well as their relation with the modulation of the gut microbiota.

## 4. Conclusions

In conclusion, given the correlations of different OTUs to the different treatments (Figure 3) in this pilot study, specific modulation of the gut microbiota towards increase or decrease of specific taxa is possible. This will be further tested in a 4-week feeding trial with these interventions.

## Data Availability

The datasets obtained and/or analyzed during the current study will be available from the corresponding author upon reasonable request.

## Supplementary Materials

Supplementary Materials Figure S1: hierarchical clustering of (a) samples at baseline (red) plus after 1-week treatment (blue) and (b) samples after 1-week treatment coded by treatment (AIN: blue; AIN_beet_juice: dark orange; AIN_psicose: yellow; Modif: grey; Modif_absorb_beet: light green; Modif_and_beet: purple; Native: dark green; Native_absorb_beet: light orange; Native_and_beet: bright red; probiotic: dark red) and whether their diets contained starch (black) or not (white). Table S1: OTUs that are significantly different between baseline (week 0) and week 1 (Kruskal-Wallis correlation; only OTUs with *q* < 0.05 are listed). Table S2: OTUs that are significantly different between treatments at week 1 (Kruskal-Wallis correlation; only OTUs with *q* < 0.05 are listed). The distribution over the different treatments is displayed in Figure 3. Table S3: OTUs that are significantly correlated to body weight or fasting plasma glucose (Kruskal-Wallis correlation; only OTUs with *q* < 0.05 are listed). The sign of the rho-values indicated positive or negative correlation.
