# Supplementary material for: Effect of Taro Starch, Beet Juice, Probiotic, and/or Psicose on Gut Microbiota in a Type 2 Diabetic Rat Model: A Pilot Study
Source: J Nutr Metab. 2021 May 20;2021:1825209. doi: 10.1155/2021/1825209 (PMC8163543; doi:10.1155/2021/1825209)
Supplement: Supplementary Materials — Figure S1: hierarchical clustering of (a) samples at baseline (red) plus after 1-week treatment (blue) and (b) samples after 1-week treatment coded by treatment (AIN: blue; AIN_beet_juice: dark orange; AIN_psicose: yellow; Modif: grey; Modif_absorb_beet: light green; Modif_and_beet: purple; Native: dark green; Native_absorb_beet: light orange; Native_and_beet: bright red; probiotic: dark red) and whether their diets contained starch (black) or not (white). Table S1: OTUs that are significantly different between baseline (week 0) and week 1 (Kruskal-Wallis correlation; only OTUs with q < 0.05 are listed). Table S2: OTUs that are significantly different between treatments at week 1 (Kruskal-Wallis correlation; only OTUs with q < 0.05 are listed). The distribution over the different treatments is displayed in Figure 3. Table S3: OTUs that are significantly correlated to body weight or fasting plasma glucose (Kruskal-Wallis correlation; only OTUs with q < 0.05 are listed). The sign of the rho-values indicated positive or negative correlation. [file 1825209.f1.zip › 1825209.f1.docx]

Table S1. OTUs that are significantly different between baseline (week 0) and week 1 (Kruskal-Wallis correlation; only OTUs with q < 0.05 are listed).

| p-value | q-value | OTU |
| --- | --- | --- |
| 1.04E-09 | 2.78E-08 | Actinobacteria;c__Actinobacteria;o__Actinomycetales;f__Corynebacteriaceae;g__Corynebacterium |
| 8.52E-10 | 2.78E-08 | Actinobacteria;c__Actinobacteria;o__Bifidobacteriales;f__Bifidobacteriaceae;g__Bifidobacterium |
| 1.1E-09 | 2.78E-08 | Actinobacteria;c__Coriobacteriia;o__Coriobacteriales;f__Coriobacteriaceae;g__ |
| 7.21E-10 | 2.78E-08 | Firmicutes;c__Bacilli;o__Bacillales;f__Bacillaceae;g__Bacillus |
| 4.07E-09 | 8.25E-08 | Firmicutes;c__Clostridia;o__Clostridiales;f__Eubacteriaceae;g__Anaerofustis |
| 4.37E-09 | 8.25E-08 | TM7;c__TM7-3;o__CW040;f__F16;g__ |
| 6.52E-08 | 9.85E-07 | Proteobacteria;c__Deltaproteobacteria;o__Desulfovibrionales;f__Desulfovibrionaceae;g__Desulfovibrio |
| 9.71E-08 | 1.33E-06 | Cyanobacteria;c__4C0d-2;o__YS2;f__;g__ |
| 3.12E-07 | 3.92E-06 | Proteobacteria;c__Betaproteobacteria;o__Burkholderiales;f__Comamonadaceae;g__Delftia |
| 3.99E-07 | 4.64E-06 | Actinobacteria;c__Actinobacteria;o__Actinomycetales;f__Streptomycetaceae;g__Streptomyces |
| 6.09E-07 | 6.57E-06 | Proteobacteria;c__Betaproteobacteria;o__Neisseriales;f__Neisseriaceae;g__ |
| 9.55E-07 | 9.09E-06 | Firmicutes;c__Clostridia;o__Clostridiales;f__Lachnospiraceae;Other |
| 9.63E-07 | 9.09E-06 | Proteobacteria;c__Alphaproteobacteria;o__Rickettsiales;f__;g__ |
| 1.24E-06 | 1.1E-05 | Firmicutes;c__Clostridia;o__Clostridiales;f__Lachnospiraceae;g__Dorea |
| 2.26E-06 | 1.9E-05 | Actinobacteria;c__Actinobacteria;o__Actinomycetales;f__Actinomycetaceae;g__Actinomyces |
| 2.4E-06 | 1.91E-05 | Firmicutes;c__Bacilli;o__Bacillales;f__Bacillaceae;g__ |
| 4.07E-06 | 3.07E-05 | Tenericutes;c__Mollicutes;o__RF39;f__;g__ |
| 4.59E-06 | 3.3E-05 | Actinobacteria;c__Actinobacteria;o__Actinomycetales;f__Dermabacteraceae;g__Brachybacterium |
| 1.49E-05 | 0.000102 | Firmicutes;c__Bacilli;o__Bacillales;f__Planococcaceae;g__Sporosarcina |
| 1.94E-05 | 0.000127 | Proteobacteria;c__Deltaproteobacteria;o__Desulfovibrionales;f__Desulfovibrionaceae;g__ |
| 2.73E-05 | 0.000172 | Proteobacteria;c__Gammaproteobacteria;o__Enterobacteriales;f__Enterobacteriaceae;g__Proteus |
| 2.94E-05 | 0.000177 | Firmicutes;c__Clostridia;o__Clostridiales;f__Veillonellaceae;g__Phascolarctobacterium |
| 3.39E-05 | 0.000197 | Actinobacteria;c__Actinobacteria;o__Actinomycetales;f__Pseudonocardiaceae;g__Saccharopolyspora |
| 5.31E-05 | 0.000297 | Proteobacteria;c__Gammaproteobacteria;o__Enterobacteriales;f__Enterobacteriaceae;Other |
| 6.34E-05 | 0.000342 | Bacteroidetes;c__Bacteroidia;o__Bacteroidales;f__Porphyromonadaceae;g__Parabacteroides |
| 8.04E-05 | 0.000405 | Firmicutes;c__Clostridia;o__Clostridiales;Other;Other |
| 8.02E-05 | 0.000405 | Firmicutes;c__Clostridia;o__Clostridiales;f__Clostridiaceae;g__SMB53 |
| 0.000159 | 0.000773 | Firmicutes;c__Clostridia;o__Clostridiales;f__Christensenellaceae;g__ |
| 0.000172 | 0.000811 | Actinobacteria;c__Actinobacteria;o__Actinomycetales;f__Microbacteriaceae;g__Curtobacterium |
| 0.000206 | 0.000942 | Proteobacteria;c__Epsilonproteobacteria;o__Campylobacterales;f__Helicobacteraceae;g__Helicobacter |
| 0.000235 | 0.001043 | Actinobacteria;c__Actinobacteria;o__Actinomycetales;f__Yaniellaceae;g__Yaniella |
| 0.000258 | 0.001115 | Firmicutes;c__Bacilli;o__Bacillales;f__Thermoactinomycetaceae;g__ |
| 0.000313 | 0.001311 | Firmicutes;c__Clostridia;o__Clostridiales;f__Veillonellaceae;g__Anaerovibrio |
| 0.000364 | 0.001484 | Firmicutes;c__Bacilli;o__Lactobacillales;f__Lactobacillaceae;Other |
| 0.000393 | 0.001561 | Firmicutes;c__Clostridia;o__Clostridiales;f__Peptococcaceae;g__rc4-4 |
| 0.000709 | 0.002745 | Firmicutes;c__Erysipelotrichi;o__Erysipelotrichales;f__Erysipelotrichaceae;g__p-75-a5 |
| 0.000764 | 0.002815 | Actinobacteria;c__Actinobacteria;o__Bifidobacteriales;f__Bifidobacteriaceae;g__ |
| 0.000759 | 0.002815 | Proteobacteria;c__Betaproteobacteria;o__Rhodocyclales;f__Rhodocyclaceae;g__Dechloromonas |
| 0.000848 | 0.003048 | Bacteroidetes;c__Bacteroidia;o__Bacteroidales;f__[Barnesiellaceae];g__ |
| 0.000951 | 0.003264 | Actinobacteria;c__Actinobacteria;o__Actinomycetales;f__Micrococcaceae;g__Rothia |
| 0.000938 | 0.003264 | Actinobacteria;c__Actinobacteria;o__Actinomycetales;f__Mycobacteriaceae;g__Mycobacterium |
| 0.000974 | 0.003267 | Firmicutes;c__Clostridia;o__Clostridiales;f__Dehalobacteriaceae;g__Dehalobacterium |
| 0.001354 | 0.004443 | Firmicutes;c__Clostridia;o__Clostridiales;f__Clostridiaceae;g__ |
| 0.001439 | 0.004622 | Firmicutes;c__Bacilli;o__Bacillales;Other;Other |

| p-value | q-value | OTU |
| --- | --- | --- |
| 2.35E-08 | 3.542E-06 | Firmicutes;c__Bacilli;o__Lactobacillales;f__Streptococcaceae;g__Lactococcus |
| 1.03E-05 | 0.000518 | Firmicutes;c__Clostridia;o__Clostridiales;f__Eubacteriaceae;g__Pseudoramibacter_Eubacterium |
| 1.89E-05 | 0.000713 | Actinobacteria;c__Coriobacteriia;o__Coriobacteriales;f__Coriobacteriaceae;g__ |
| 2.62E-05 | 0.000793 | Actinobacteria;c__Actinobacteria;o__Bifidobacteriales;f__Bifidobacteriaceae;g__ |
| 5.25E-05 | 0.001321 | Firmicutes;c__Bacilli;o__Lactobacillales;f__Leuconostocaceae;g__Leuconostoc |
| 8.05E-05 | 0.001736 | Firmicutes;c__Clostridia;o__Clostridiales;f__Peptococcaceae;g__rc4-4 |
| 0.000106 | 0.001789 | Actinobacteria;c__Actinobacteria;o__Bifidobacteriales;f__Bifidobacteriaceae;g__Bifidobacterium |
| 9.85E-05 | 0.001789 | Firmicutes;c__Clostridia;o__Clostridiales;f__Veillonellaceae;g__Anaerovibrio |
| 0.000205 | 0.003096 | Proteobacteria;c__Deltaproteobacteria;o__Desulfovibrionales;f__Desulfovibrionaceae;g__Desulfovibrio |
| 0.000303 | 0.003815 | Proteobacteria;c__Gammaproteobacteria;o__Enterobacteriales;f__Enterobacteriaceae;Other |
| 0.000429 | 0.004746 | Firmicutes;c__Clostridia;o__Clostridiales;f__Christensenellaceae;g__ |
| 0.000440 | 0.004746 | Firmicutes;c__Clostridia;o__Clostridiales;f__Veillonellaceae;g__Phascolarctobacterium |
| 0.000486 | 0.004893 | Firmicutes;c__Bacilli;o__Bacillales;f__Bacillaceae;g__Bacillus |

Table S2. OTUs that are significantly different between treatments at week 1 (Kruskal-Wallis correlation; only OTUs with q < 0.05 are listed). The distribution over the different treatments is displayed in Figure 3.

| p-value | q-value | rho-value | OTU |
| --- | --- | --- | --- |
| bodyweight | | |  |
| 0.00043 | 0.0324 | 0.39 | Proteobacteria;c__Alphaproteobacteria;o__Rhizobiales;Other;Other |
| 0.00030 | 0.0324 | -0.40 | Proteobacteria;c__Deltaproteobacteria;o__Desulfovibrionales;f__Desulfovibrionaceae;  g__Desulfovibrio |
| 0.00175 | 0.04939 | -0.35 | Actinobacteria;c__Actinobacteria;o__Actinomycetales;f__Micrococcaceae;g__Rothia |
| 0.00196 | 0.04939 | -0.35 | Actinobacteria;c__Actinobacteria;o__Actinomycetales;f__Streptomycetaceae;g__Streptomyces |
| 0.00143 | 0.04939 | -0.36 | Bacteroidetes;c__Bacteroidia;o__Bacteroidales;f__RF16;g__ |
| 0.00189 | 0.04939 | 0.35 | Firmicutes;c__Bacilli;o__Bacillales;f__Planococcaceae;g__ |
| fasting plasma glucose | | |  |
| 0.000256 | 0.038639 | 0.41 | Actinobacteria;c__Actinobacteria;o__Actinomycetales;f__Micrococcaceae;g__Rothia |

Table S3. OTUs that are significantly correlated to body weight or fasting plasma glucose (Kruskal-Wallis correlation; only OTUs with q < 0.05 are listed). The sign of the rho-values indicated positive or negative correlation.
