# Supplementary figures and images for: Effect of Taro Starch, Beet Juice, Probiotic, and/or Psicose on Gut Microbiota in a Type 2 Diabetic Rat Model: A Pilot Study
Source: J Nutr Metab. 2021 May 20;2021:1825209. doi: 10.1155/2021/1825209 (PMC8163543; doi:10.1155/2021/1825209)

## Slide 1
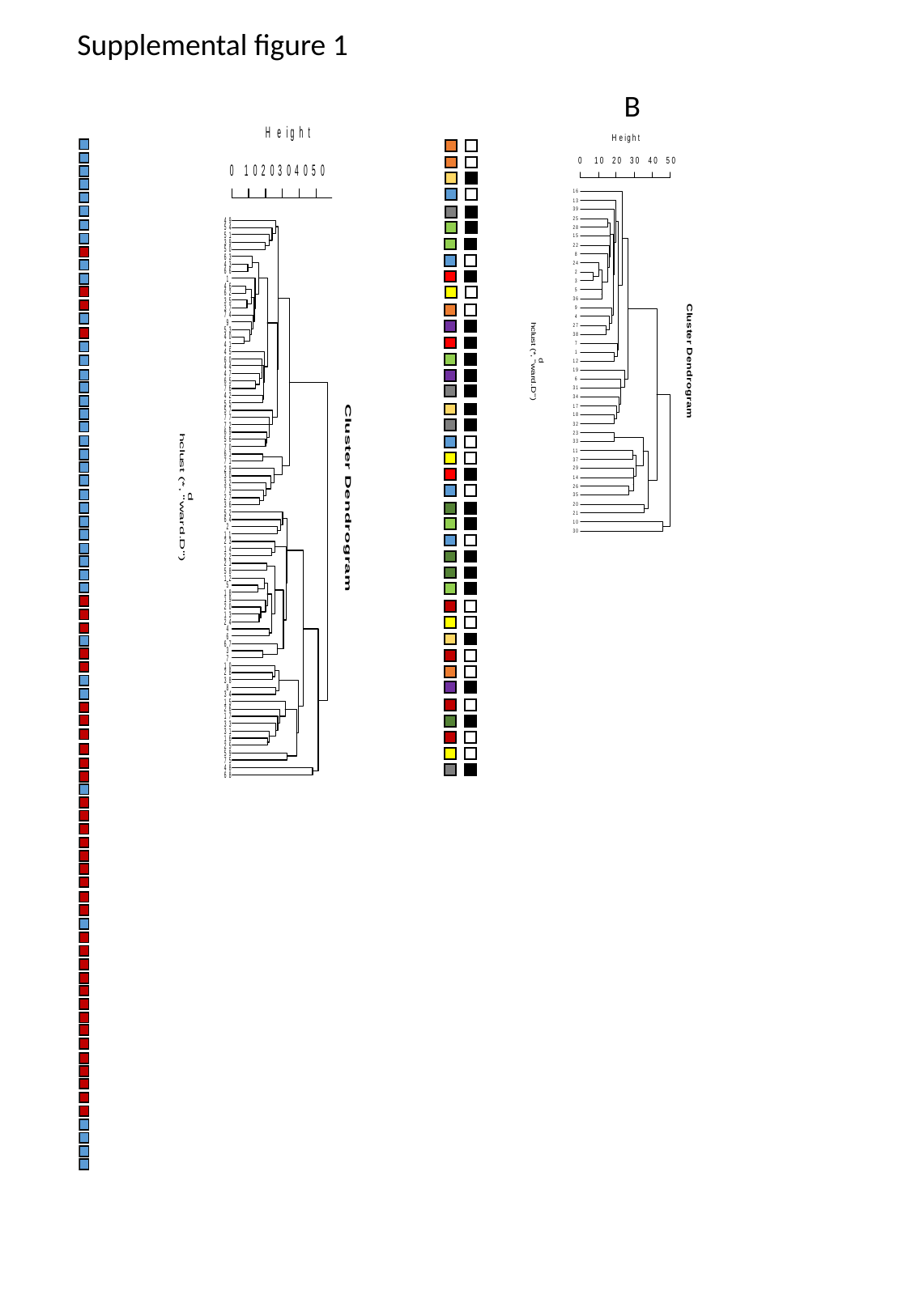

Supplemental figure 1
A						B

Supplement: Supplementary Materials — Figure S1: hierarchical clustering of (a) samples at baseline (red) plus after 1-week treatment (blue) and (b) samples after 1-week treatment coded by treatment (AIN: blue; AIN_beet_juice: dark orange; AIN_psicose: yellow; Modif: grey; Modif_absorb_beet: light green; Modif_and_beet: purple; Native: dark green; Native_absorb_beet: light orange; Native_and_beet: bright red; probiotic: dark red) and whether their diets contained starch (black) or not (white). Table S1: OTUs that are significantly different between baseline (week 0) and week 1 (Kruskal-Wallis correlation; only OTUs with q < 0.05 are listed). Table S2: OTUs that are significantly different between treatments at week 1 (Kruskal-Wallis correlation; only OTUs with q < 0.05 are listed). The distribution over the different treatments is displayed in Figure 3. Table S3: OTUs that are significantly correlated to body weight or fasting plasma glucose (Kruskal-Wallis correlation; only OTUs with q < 0.05 are listed). The sign of the rho-values indicated positive or negative correlation. [file 1825209.f1.zip › Supplemental Figure 1.pptx]
